# Development and validation of the predictive risk of death model for adult patients admitted to intensive care units in Japan: an approach to improve the accuracy of healthcare quality measures

**DOI:** 10.1186/s40560-021-00533-z

**Published:** 2021-02-15

**Authors:** Hideki Endo, Shigehiko Uchino, Satoru Hashimoto, Yoshitaka Aoki, Eiji Hashiba, Junji Hatakeyama, Katsura Hayakawa, Nao Ichihara, Hiromasa Irie, Tatsuya Kawasaki, Junji Kumasawa, Hiroshi Kurosawa, Tomoyuki Nakamura, Hiroyuki Ohbe, Hiroshi Okamoto, Hidenobu Shigemitsu, Takashi Tagami, Shunsuke Takaki, Kohei Takimoto, Masatoshi Uchida, Hiroaki Miyata

**Affiliations:** 1 Department of Healthcare Quality Assessment, Graduate School of Medicine, The University of Tokyo, 7-3-1 Hongo, Bunkyo-ku, Tokyo, 113-8655 Japan; 2 Department of Health Policy and Management, School of Medicine, Keio University, 35 Shinanomachi, Shinjuku-ku, Tokyo, 160-8582 Japan; 3 Intensive Care Unit, The Jikei University School of Medicine, 3-19-18 Nishi-Shinbashi, Minato-ku, Tokyo, 105-8471 Japan; 4 Department of Anesthesiology and Intensive Care Medicine, Kyoto Prefectural University of Medicine, 465 Kajii-cho, Kawaramachi-Hirokoji, Kamigyo-ku, Kyoto, 602-8566 Japan; 5 Department of Anesthesiology and Intensive Care Medicine, Hamamatsu University School of Medicine, 1-20-1 Handayama, Higashi-ku, Hamamatsu, Shizuoka, 431-3192 Japan; 6 Division of Intensive Care, Hirosaki University Hospital, 53 Honcho, Hirosaki, Aomori, 036-8203 Japan; 7 Department of Emergency and Critical Care Medicine, National Hospital Organization Tokyo Medical Center, 2-5-1, Higashigaoka, Meguro-ku, Tokyo, 152-8902 Japan; 8 Department of Emergency and Critical Care Medicine, Saitama Red Cross Hospital, 1-5 Shintoshin, Chuo-ku, Saitama, 330-8553 Japan; 9 Department of Anesthesiology, Kurashiki Central Hospital, 1-1-1 Miwa, Kurashiki, Okayama, 710-8602 Japan; 10 Department of Pediatric Critical Care, Shizuoka Children’s Hospital, 860 Urushiyama, Aoi-ku, Shizuoka, Shizuoka 420-8660 Japan; 11 Department of Critical Care Medicine, Sakai City Medical Center, 1-1-1 Ebaraji-cho, Nishi-ku, Sakai, Osaka, 593-8304 Japan; 12 Department of Pediatric Critical Care Medicine, Hyogo Prefectural Kobe Children’s Hospital, 1-6-7 Minatojima Minamimachi, Chuo-ku, Kobe, Hyogo 650-0047 Japan; 13 Department of Anesthesiology and Critical Care Medicine, Fujita Health University School of Medicine, 1-98 Dengakugakubo, Kutsukake-cho, Toyoake, Aichi 470-1192 Japan; 14 Department of Clinical Epidemiology and Health Economics, School of Public Health, The University of Tokyo, 7-3-1 Hongo, Bunkyo-ku, Tokyo, 113-0033 Japan; 15 Department of Critical Care Medicine, St. Luke’s International Hospital, 9-1 Akashi-cho, Chuo-ku, Tokyo, 104-8560 Japan; 16 Department of Intensive Care Medicine, Graduate School of Medicine, Tokyo Medical and Dental University, 1-5-45 Yushima, Bunkyo-ku, Tokyo, 113-8519 Japan; 17 Department of Emergency and Critical Care Medicine, Nippon Medical School Musashikosugi Hospital, 1-396 Kosugimachi, Nakahara-ku, Kawasaki, Kanagawa 211-8533 Japan; 18 Department of Anesthesiology and Critical Care Medicine, Yokohama City University, 3-9 Fukuura, Kanazawa-ku, Yokohama, Kanagawa 236-0004 Japan; 19 Department of Intensive Care Medicine, Kameda Medical Center, 929 Higashi-cho, Kamogawa, Chiba, 296-8602 Japan; 20 Department of Emergency and Critical Care Medicine, Dokkyo Medical University, 880 Kitakobayashi, Mibu-machi, Shimotsuga-gun, Tochigi, 321-0293 Japan

**Keywords:** Risk of death, Risk prediction model, Recalibration, Benchmarking, Quality improvement, Quality indicator

## Abstract

**Background:**

The Acute Physiology and Chronic Health Evaluation (APACHE) III-j model is widely used to predict mortality in Japanese intensive care units (ICUs). Although the model’s discrimination is excellent, its calibration is poor. APACHE III-j overestimates the risk of death, making its evaluation of healthcare quality inaccurate. This study aimed to improve the calibration of the model and develop a Japan Risk of Death (JROD) model for benchmarking purposes.

**Methods:**

A retrospective analysis was conducted using a national clinical registry of ICU patients in Japan. Adult patients admitted to an ICU between April 1, 2018, and March 31, 2019, were included. The APACHE III-j model was recalibrated with the following models: Model 1, predicting mortality with an offset variable for the linear predictor of the APACHE III-j model using a generalized linear model; model 2, predicting mortality with the linear predictor of the APACHE III-j model using a generalized linear model; and model 3, predicting mortality with the linear predictor of the APACHE III-j model using a hierarchical generalized additive model. Model performance was assessed with the area under the receiver operating characteristic curve (AUROC), the Brier score, and the modified Hosmer–Lemeshow test. To confirm model applicability to evaluating quality of care, funnel plots of the standardized mortality ratio and exponentially weighted moving average (EWMA) charts for mortality were drawn.

**Results:**

In total, 33,557 patients from 44 ICUs were included in the study population. ICU mortality was 3.8%, and hospital mortality was 8.1%. The AUROC, Brier score, and modified Hosmer–Lemeshow *p* value of the original model and models 1, 2, and 3 were 0.915, 0.062, and < .001; 0.915, 0.047, and < .001; 0.915, 0.047, and .002; and 0.917, 0.047, and .84, respectively. Except for model 3, the funnel plots showed overdispersion. The validity of the EWMA charts for the recalibrated models was determined by visual inspection.

**Conclusions:**

Model 3 showed good performance and can be adopted as the JROD model for monitoring quality of care in an ICU, although further investigation of the clinical validity of outlier detection is required. This update method may also be useful in other settings.

**Supplementary Information:**

The online version contains supplementary material available at 10.1186/s40560-021-00533-z.

## Background

Various risk prediction models for intensive care unit (ICU) patients have been developed to predict an individual patient’s risk of death during hospitalization and to monitor the quality of care by comparing predicted and observed mortality [1]. The Acute Physiology and Chronic Health Evaluation (APACHE), the Mortality Probability Model, and the Simplified Acute Physiology Score are widely used models that have been updated several times to improve their performance [2, 3]. The Australian and New Zealand Intensive Care Society (ANZICS) and the Intensive Care National Audit & Research Centre (ICNARC) have updated their own risk prediction models for better model performance [4, 5]. The risk model should be adjusted to the population and clinical environment of a particular setting.

The APACHE III-j is a risk prediction model for in-hospital mortality that is widely used in Japanese ICUs. However, APACHE III-j overestimated the risk of death for Japanese ICU patients, producing estimates that are more than two times the observed mortality [6]. It is imperative to have an accurate risk prediction model to appraise individual risk of death in daily clinical practice and evaluate the overall quality of care in an ICU using quality metrics such as the standardized mortality ratio (SMR), a ratio of observed mortality to expected mortality [7]. For example, the ANZICS developed and validated their own risk prediction model, the Australian and New Zealand Risk of Death model, to improve benchmarking performance [4, 8].

The aim of this study was to update the APACHE III-j model and optimize it for use in the Japanese ICU setting. The newly developed and validated model—the Japan Risk of Death (JROD) model—was designed to be used for benchmarking and tracking quality measures. We tested the applicability of the updated model to quality indicators with SMR funnel plots and exponentially weighted moving average (EWMA) charts for mortality. These two graphical tools have also been used in quality reports distributed by the ANZICS and the ICNARC [5, 9].

## Methods

### Data collection

We obtained data from the Japanese Intensive care PAtient Database (JIPAD), the largest clinical registry of ICU patients (both adults and children) in Japan. It is governed by the ICU Functional Assessment Committee of the Japanese Society for Intensive Care Medicine. As of October 2020, 77 ICUs participate in the JIPAD, and the database includes information on more than 170,000 patients. The JIPAD works in partnership with and collects similar data to the ANZICS database, including the clinical severity information needed to calculate risk of death using the APACHE II, APACHE III, Simplified Acute Physiology Score II, Paediatric Index of Mortality 2, and Paediatric Index of Mortality 3 models. To improve the accuracy of data entry, the quality of data entry is checked when ICUs begin to participate in the JIPAD and routinely thereafter by members of the JIPAD Working Group. Participating ICUs submit data on all patients admitted to their ICUs in each fiscal year. More details on the JIPAD can be found elsewhere [6].

This study was approved by the Research Ethics Committee of the University of Tokyo (Approval number: 2020242NI). The need to collect informed consent was waived because all data were handled in an anonymized fashion.

### Study population

Patients who were admitted to the ICU from April 1, 2018, to March 31, 2019, were included in the study. Because our aim was to update the APACHE III-j model, a risk prediction model for adults [10], patients who were admitted to a pediatric ICU or were younger than 16 years were excluded from the study population. Patients who were readmitted to the ICU during the same hospitalization were also excluded. Additionally, patients who were only admitted to the ICU for a single medical procedure such as central venous catheterization or cardioversion for atrial fibrillation were excluded because they were not considered as receiving “intensive” care. Finally, patients who had missing values on discharge outcome were excluded from the analysis.

### Recalibration methods

Because the discrimination of the APACHE III-j model has been shown to be excellent in the Japanese ICU population [6], our focus in developing a new model was to improve the calibration of the APACHE III-j model. The predicted mortality needed to be reduced. Motivated by the recalibration methods of Steyerberg [11], we began by fitting two models. Model 1 predicted in-hospital mortality using an offset variable (i.e., a coefficient fixed at 1) for the linear predictor of the APACHE III-j model as the only explanatory variable. This model updates only the intercept of the original model. This update accounts for the change in the case mix of the population and improves the model such that the mean predicted mortality equals the mean observed mortality [11]. The equation for calculating the log odds of the probability of death is

$$ \mathrm{Logit}={\beta}_0+\mathrm{offset}\left(\mathrm{lp}\right), $$

where *β*_0_ is the intercept and lp is the linear predictor of the APACHE III-j model. Because we had the APACHE III-j predicted mortality in our database, lp was calculated as

$$ \mathrm{lp}=\log \left(\frac{\mathrm{APACHEIII}-\mathrm{jpredictedmortality}}{1-\mathrm{APACHEIII}-\mathrm{jpredictedmortality}}\right). $$

Model 2 incorporated the only explanatory variable in model 1 as an ordinary variable (i.e., a variable without an offset). This model updates the overall coefficient of the linear predictor of the APACHE III-j model in addition to the intercept and intends to achieve a calibration plot with a slope of 1 and an intercept of 0 [11]. The model is calculated as

$$ \mathrm{Logit}={\beta}_0+{\beta}_1\times \mathrm{lp}, $$

where *β*_1_ is the coefficient of the linear predictor. Using these recalibration methods, we evaluated the model performance and applicability for monitoring quality of care.

### Model performance assessment

Model performance was assessed using the area under the receiver operating characteristic curve (AUROC) [12], the Brier score [13], the scaled Brier score [14], the modified Hosmer–Lemeshow test [15], the calibration plot [14], and the Akaike information criterion (AIC) [16]. The AUROC is a test for discrimination, the Brier score and the scaled Brier score are used to test both discrimination and calibration, and the modified Hosmer–Lemeshow test and the calibration plot are used to test calibration. The AIC was used to compare the different models’ fit to the data. The Brier score is an average of the squares of the differences between the predicted probability of the outcome and the observed outcome. It ranges from 0 and 1, and smaller values indicate better model performance. The scaled Brier score is an adjustment of the Brier score to mitigate the influence of the proportion of the population experiencing the outcome on the score. It can be interpreted similarly to Pearson’s *R*^*2*^ [14]. The modified Hosmer–Lemeshow test is a modified version of the Hosmer–Lemeshow test that can be used for large datasets. The original Hosmer–Lemeshow test is overpowered when the sample size is large, leading to a rejection of the null hypothesis of no difference between the observed and expected proportions of the outcome. The calibration plot is a visual aid for assessing calibration. Here, observed mortality is plotted against predicted mortality. Values for the slope and intercept of 1 and 0, respectively, indicate perfect calibration. For both the modified Hosmer–Lemeshow test and the calibration plot, following common practice in the field, the study population was divided into deciles according to the predicted mortality. Finally, the AIC is a relative measure that evaluates model fit and penalizes overfit [16]. The AIC is calculated as negative two times the log likelihood plus two times the parameters to be estimated. Lower AIC values indicate better model fit to the data.

### Practical applicability to quality metrics

The applicability of the models was checked with SMR funnel plots and EWMA charts for mortality. Funnel plots have been used to compare quality across facilities [17, 18]. Here, the quality indicators are plotted against a precision parameter (e.g., the expected number of deaths), and the control limits present a funnel-like shape indicating whether the quality indicator is “in control” or “out of control.” Plots inside the funnel are considered “in control.” In our study, the SMR was chosen as the quality indicator, and the expected number of deaths determined using each model was selected as the precision parameter. The variance of the plots was checked with overdispersion factor *Φ* because the variance may exceed the degree of random variation, and overdispersion may indicate model misspecification [19]. *Φ* is calculated as the sum of the squared standardized Pearson residuals of each plot divided by the number of sample units (i.e., the number of ICUs) [17, 18]. *Φs* larger than 1 were considered to indicate overdispersion [17, 18]. EWMA charts plot the moving average of a quality indicator, with certain weights assigned to the latest and earlier data [20]. Lambda, which is the weight assigned to the latest data in EWMA charts, was set at 0.005 [9], and the mean mortality was chosen as the starting point of the chart. Control limits calculated from the expected mortality were drawn to evaluate “in control” moves. Funnel plots summarize quality as a single point during a certain time period, whereas EWMA charts are graphs that capture the dynamic trends in quality indicators sequentially. EWMA charts are more informative than funnel plots because they can capture abnormal trends and spot deviations.

### Improving the recalibration method

After these evaluations, another method was employed to improve the model’s calibration. We assumed that the calibration plot could be improved further to move closer to the diagonal line. The results of the modified Hosmer–Lemeshow test also indicated that the calibration was still poor. We also considered that overdispersion could be alleviated in the funnel plot. For model 3, we updated model 2 with a hierarchical generalized additive model [21]. The generalized additive model is an extension of the generalized linear model with greater flexibility in modeling the associations between the outcome and the explanatory variables. The advantage of the model is that it can model nonlinear relationships. Further, we adopted a hierarchical model to deal with overdispersion in the funnel plots, where many data values lying outside the control limits may mean the model is less useful for detecting real outliers [19, 22]. Hierarchical models incorporate cluster-level variance, which reduces overdispersion. A random intercept for ICUs was added to the generalized additive model to account for ICU-level characteristics. The model can be written as

$$ \mathrm{Logit}={\beta}_0+f\left(\mathrm{lp}\right)+{u}_i $$

$$ {u}_i\sim N\left(0,{\sigma}^2\right), $$

where *f* is a smooth function for modeling a nonlinear relationship and *u*_*i*_ is a random intercept for ICU *i*, which has a normal distribution with a mean of 0 and variance of *σ*^2^.

### Model validation

The optimism-corrected AUROC was calculated to evaluate the validity of the models [23]. It is possible that the developed models overfit the study population data and that the model performance values are too good (i.e., “over-optimistic”). The optimism-corrected AUROC was used to assess whether overfitting occurred and to reevaluate the AUROC without the effect of over-optimism. The optimism-corrected AUROC was calculated as follows: the AUROC in the original study population minus average optimism, where optimism is the AUROC in a bootstrap sample of the study population minus the tested AUROC in the original study population. The optimism-corrected AUROCs for models 1 and 2 were calculated with 5000 bootstrap samples. For model 3, the optimism-corrected AUROC was calculated from a posterior distribution of 10,000 simulated model samples [21].

### Statistical analysis

A *p* value of ≤ .05 was considered statistically significant in the modified Hosmer–Lemeshow tests and calibration plots. The three-standard deviation threshold, conventional as a control limit in quality control, was used to determine extreme cases in the funnel plots and EWMA charts [17, 20]. We used R version 3.6.3 (2020; R Foundation for Statistical Computing, Vienna, Austria) for all statistical analyses. The mgcv package version 1.8–33 was used to compute the hierarchical generalized additive model. The R code for fitting models 1, 2, and 3 is provided in Additional file 1.

## Results

**Fig. 1:**
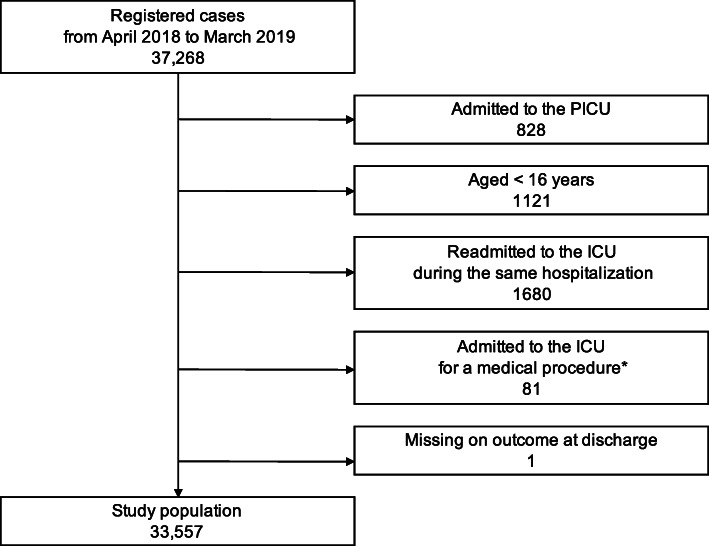
Study population flowchart. *Procedures such as central venous catheterization and cardioversion for atrial fibrillation. ICU, intensive care unit; PICU, pediatric intensive care unit

A total of 33,557 patients from 44 ICUs were included in the study population (Fig. 1). Of these ICUs, 21 were in university hospitals. ICU mortality was 3.8%, and hospital mortality was 8.1 (Table 1). Nearly 60% of the admissions were for elective surgery, and cardiovascular diseases were listed as the primary diagnosis for more than one third of the admissions.

**Table 1:** Patient characteristics and outcomes

Clinical characteristic	Number
Number of patients	33,557
Age, mean (SD)	67.6 (15.0)
Male	20,515 (61.1%)
Elective admission	19,649 (58.6%)
Admission classification	
Elective surgery	19,774 (58.9%)
Emergency surgery	4343 (12.9%)
Non-surgical	9440 (28.1%)
Admission source	
Operating room	23,272 (69.4%)
Emergency room	6792 (20.2%)
Hospital ward	2728 (8.1%)
Other ICU, same hospital	95 (0.3%)
Coronary care unit	21 (0.1%)
High care unit	333 (1.0%)
Other hospital	315 (0.9%)
Chronic illness	
Immunosuppression	1862 (5.5%)
Maintenance dialysis	1610 (4.8%)
Metastatic tumor	1363 (4.1%)
Respiratory failure	331 (1.0%)
Liver cirrhosis	392 (1.2%)
Heart failure	412 (1.2%)
Acute leukemia/Multiple myeloma	181 (0.5%)
Lymphoma	201 (0.6%)
Liver failure	149 (0.4%)
AIDS	20 (0.1%)
Disease category	
Cardiovascular	11,694 (34.8%)
Neurological	4664 (13.9%)
Respiratory	4940 (14.7%)
Gastrointestinal	6579 (19.6%)
Musculoskeletal	1277 (3.8%)
Genitourinary	1292 (3.9%)
Trauma	773 (2.3%)
Metabolic	778 (2.3%)
Hematological	120 (0.4%)
Gynecological	771 (2.3%)
Other	669 (2.0%)
Outcome	
APACHE III score, mean (SD)	58.3 (27.9)
APACHE III-j predicted risk of death, mean % (SD)	17.0 (22.8)
Length of ICU stay, median days (IQR)	2 (2–5)
Length of hospital stay, median days (IQR)	20 (12–36)
Deaths before ICU discharge	1291 (3.8%)
Deaths before hospital discharge	2728 (8.1%)

*AIDS* acquired immunodeficiency syndrome, *APACHE* Acute Physiology and Chronic Health Evaluation, *ICU* intensive care unit, *IQR* interquartile range, *SD* standard deviation

**Fig. 2:**
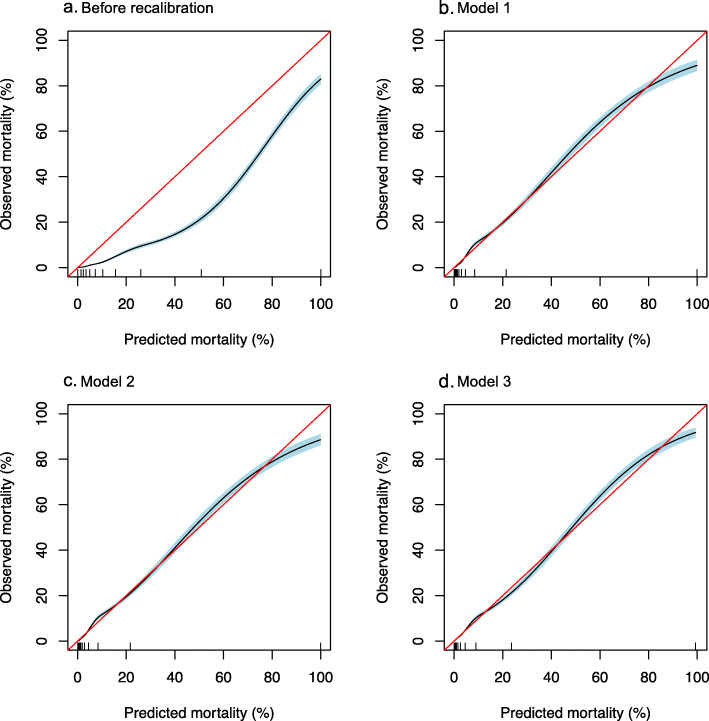
Calibration plots. A natural spline was used to draw the plots with a shaded area indicating the 95% confidence interval. Ideally, the calibration plot aligns with the diagonal line (in red). A rug plot is presented along the *x*-axis. Model 1 predicted in-hospital mortality with an offset variable for the linear predictor of the APACHE III-j model using a generalized linear model. Model 2 predicted in-hospital mortality with the linear predictor of the APACHE III-j model using a generalized linear model. Model 3 predicted in-hospital mortality with the linear predictor of the APACHE III-j model using a hierarchical generalized additive model

A summary of model performance is shown in Table 2, and the coefficients of the models are provided in Additional files 1 and 2. An approximate calculation of predicted mortality for model 3 is also available in Additional file 2. The AUROCs exceeded 0.9 in all models. The Brier score and the scaled Brier score were comparable in models 1, 2, and 3. Model 3 was the only model that yielded a non-significant result for the modified Hosmer–Lemeshow test. The slope and intercept of the calibration plot in all recalibrated models were close to 1 and 0, respectively. The optimism-corrected AUROCs differed minimally from the original AUROCs in all recalibrated models. Figure 2 illustrates the calibration plots of the models; the plot of model 3 was visually determined to be the closest to the diagonal line. Model 3 had the lowest AIC among the recalibrated models.

**Table 2:** Performance of the prediction models

Model performance	Before recalibration	Model 1	Model 2	Model 3
AUROC	0.915	0.915	0.915	0.917
Optimism-corrected AUROC	-	0.915	0.915	0.916
Brier score	0.062	0.047	0.047	0.047
Scaled Brier score	0.171	0.37	0.37	0.38
Modified Hosmer–Lemeshow test, *p* value	< .001	< .001	.002	.84
Calibration plot				
Intercept	- 1.32	0.04	0.00	0.01
Slope	1.02	1.02	1.00	1.01
Akaike information criterion	-	11219	11219	11129
Standardized mortality ratio	0.48	1.00	1.00	1.00

*AUROC* area under the receiver operating characteristic curve

Model 1 predicted in-hospital mortality with an offset variable for the linear predictor of the APACHE III-j model using a generalized linear model. Model 2 predicted in-hospital mortality with the linear predictor of the APACHE III-j model using a generalized linear model. Model 3 predicted in-hospital mortality with the linear predictor of the APACHE III-j model using a hierarchical generalized additive model.

**Fig. 3:**
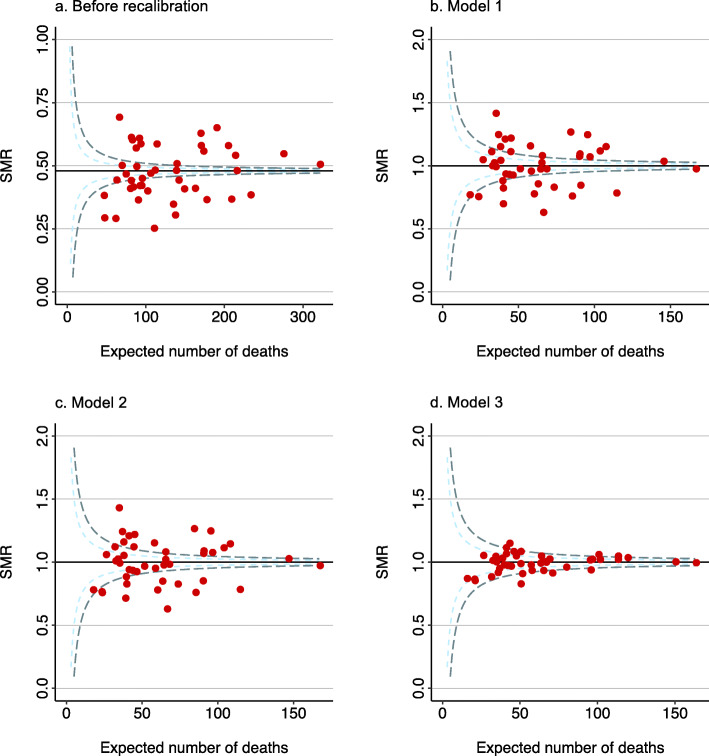
Funnel plots. The light and dark green dashed lines indicate the two- and three-standard deviation limits, respectively. The horizontal black line shows the SMR of the population. Each red dot represents the SMR of an individual intensive care unit. SMR, standardized mortality ratio. Model 1 predicted in-hospital mortality with an offset variable for the linear predictor of the APACHE III-j model using a generalized linear model. Model 2 predicted in-hospital mortality with the linear predictor of the APACHE III-j model using a generalized linear model. Model 3 predicted in-hospital mortality with the linear predictor of the APACHE III-j model using a hierarchical generalized additive model

**Fig. 4:**
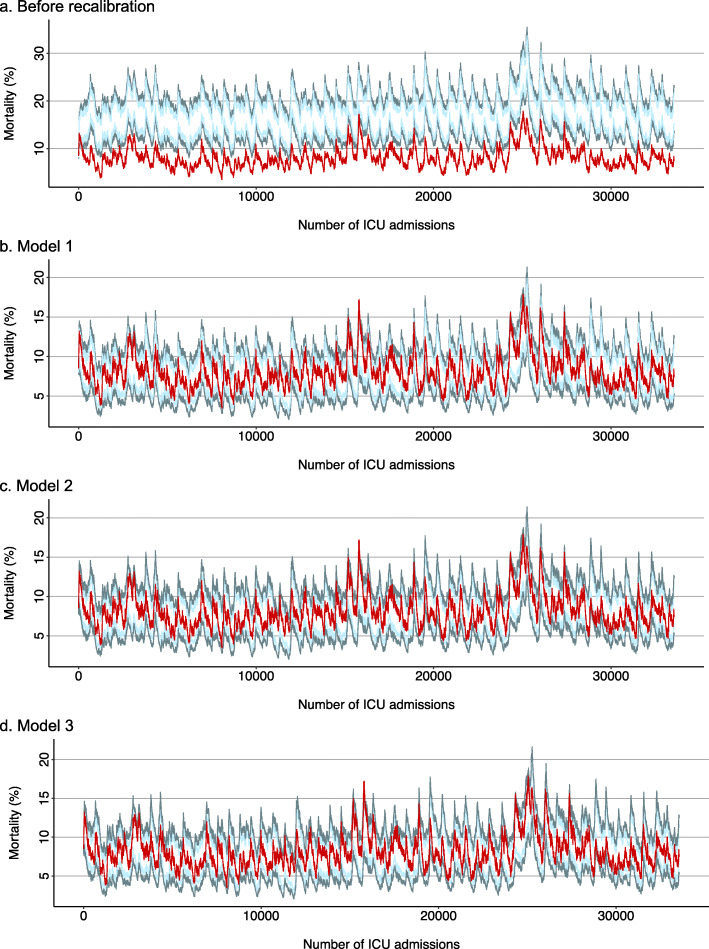
Exponentially weighted moving average charts. Sequential admissions are presented on the *x*-axis. The red lines show the exponentially weighted moving average of mortality, with the starting point of the average mortality during the study period (i.e., 8.1%) and with a weight of 0.005 on the latest data. The light and dark green lines are the control limits representing two- and three-standard deviations, respectively. ICU, intensive care unit. Model 1 predicted in-hospital mortality with an offset variable for the linear predictor of the APACHE III-j model using a generalized linear model. Model 2 predicted in-hospital mortality with the linear predictor of the APACHE III-j model using a generalized linear model. Model 3 predicted in-hospital mortality with the linear predictor of the APACHE III-j model using a hierarchical generalized additive model

The SMR funnel plots are presented in Fig. 3. Overdispersion was present, and, except for model 3, many ICUs were plotted outside the three-standard deviation limits in the recalibrated models. The mortality EWMA chart for the original model revealed a plot that moved completely outside the control limits (Fig. 4). In contrast, the charts for models 1, 2, and 3 revealed “in-control” moves and were almost identical to each other. The EWMA charts for model 3 individualized for the 44 ICUs are presented in Fig. S1 in Additional file 1. The moving average of two ICUs crossed the upper control limit of three-standard deviations.

## Discussion

We recalibrated the APACHE III-j model to improve the accuracy of predictions of the risk of death in the Japanese clinical registry of ICU patients. The discrimination of the models was excellent, with all models, including the original model, with AUROC values exceeding 0.9. The calibration improved with model recalibration; the largest improvement was achieved in Model 3, as evidenced by the Brier score, the scaled Brier score, and the calibration plot. The lowest AIC showed model 3 to be the model with the best fit to the data. Over-optimism was minimal, judging from the difference between the original and optimism-corrected AUROCs.

The funnel plots of the recalibrated models showed overdispersion, except for model 3. Clustering patients from the same ICU seemed effective to reduce overdispersion. Among the 44 ICUs, 21 were in university hospitals; therefore, variation at the ICU level may have existed. Even if the model performance is good, as seen in models 1 and 2, the model needs further adjustment to be applied to quality metrics.

The EWMA chart for the original APACHE-III-j model was “out of control” throughout the study period, reflecting an overestimation of the risk of death. The recalibrated models had seemingly reasonable EWMA charts.

Judging from these results, model 3 was the best recalibrated model and could be applied to SMR funnel plots and EWMA charts for mortality. This model can be adopted as the JROD model. When compared with the results of the Australian and New Zealand Risk of Death and ICNARC models [4, 5], our JROD model showed a similar model performance. Discrimination was excellent, and calibration was modest [11]. As the ANZICS and the ICNARC use their models for risk prediction and distribute quality reports periodically, the JROD model can be used for benchmarking and quality improvement purposes in Japan.

Because the performance of a risk prediction model deteriorates over time [24, 25], its performance should be evaluated periodically to ensure the credibility of the risk prediction. Discrimination was excellent in our study population but calibration was poor. Calibration becomes poor if the case mix of the population, observed mortality, or the quality of the data changes [26–28]. The case mix of our study population was different from that of the development population for APACHE III. For example, nearly 60% of the cases in this study were elective surgeries, which accounted for only one third of the cases in the APACHE III study [10]; this may have contributed to poor calibration. If the discriminatory power of the model is good but its calibration becomes poor, which is common in risk prediction models [24, 25], the update method used in model 3 can be easily applied to update the model. Other model update methods require extensive revisions such as updating the coefficients of all covariates and adding potentially relevant variables to the existing model [4, 5]. This extensive type of update may be necessary if the discrimination needs to be improved, but such updates require extensive computation and reevaluation. It is also possible that important variables will be degraded or even omitted at each update, especially if the sample size is small [11]. Our update method used in model 3 is an easy-to-use and valid method that we assume is sufficient for most periodic updates. Other healthcare systems with limited resources may also benefit from adopting our update method. We recommend evaluating the model’s performance periodically (e.g., when annual quality reports are made) and updating the model if needed.

The updated JROD model presented in this study may soon be out of date. In particular, the calibration of the model may deteriorate within a few years. We may need to add the year of recalibration after the model name (i.e., JROD_2018_) to ensure the year of the study population used in the recalibration process is immediately apparent. The ICNARC, for example, has named their recalibrated model ICNARC_H-2015_ [29].

There are several limitations that should be kept in mind when interpreting the results of this study. First, we performed local adjustment to the Japanese ICU population from April 2018 to March 2019. External validation was not conducted; however, this step was not necessary because increasing the generalizability of the model was not our aim. The model is intended to be used for evaluating the quality of care for patients included in the sample population. We tested the internal validity of the model, and overfitting was not apparent. Each country or healthcare system should adjust the model to their local environment.

Second, we inspected the applicability of the funnel plots and EWMA charts only statistically and graphically. We have not yet investigated the ICUs that were located outside the control limits of the funnel plots and EWMA charts. As shown in Fig. 3, eight of the 44 participating ICUs were plotted outside the control limits in the funnel plot for the JROD model. Statistically, only 0.2% of the ICUs should exceed the control limits of three-standard deviations if no special causes are present. The eight ICUs falling outside the control limits need to be explored to determine whether they are true outliers. It is also possible that the “out-of-control” signal was caused by a lack of adjustment in the case mix that was not incorporated in the model or by errors in the submitted data [20]. Although ICUs falling below the lower control limit may be reassured or satisfied with their good performance, investigating outliers that cross the upper control limit, indicating significantly worse outcomes than expected, is a sensitive issue because the quality of these ICUs will need to be evaluated using outside references. Some ICUs may have to reconsider their practice patterns, which will often impose a heavy burden on them. No one wants to have it pointed out that they are “out of control,” when every healthcare provider is fighting hard for their patients. The clinical credibility of the model should be verified so that the quality metrics can be used in quality improvement activities.

Third, modern techniques such as machine learning may improve the accuracy of prediction [30, 31]. However, how much improvement in model performance is required is unknown. The original APACHE III-j model showed excellent discriminatory power in the Japanese ICU population. Our JROD model had good calibration and no apparent overdispersion in the funnel plot; we therefore decided to evaluate the clinical validity in the next step. Moreover, the update method used in model 3 is simple and does not require much data wrangling. We assume that whether further improvement is necessary depends on how much one expects from model improvement and how many resources the working group can invest in it. The mission of the working group is to improve not only risk prediction but also quality of care. Much of the work should be dedicated to assessing the clinical validity of the model in the use of quality metrics and taking measures to improve clinical outcomes. If the clinical validity turns out to be suboptimal, we may reconsider incorporating machine learning algorithms in the JROD model.

A final limitation is that we did not assess model performance among different subgroups, such as patients with cardiac diseases as the primary reason for ICU admission. Various subgroups could be considered [32, 33] when recalibrating the model for each subgroup to obtain an accurate prediction of mortality. This would be beneficial for assessing the quality of care within specific clinical groups. Quality improvement initiatives for all ICU patients may be too broad to implement. Individualization of the JROD model for subgroups will likely be needed to instigate a quality improvement project.

## Conclusions

In summary, we were able to recalibrate the APACHE III-j model with good model performance. The model that predicted in-hospital mortality with the APACHE III-j predicted mortality using a hierarchical generalized additive model was adopted as the JROD model. This recalibration method can also be used with ease with other risk prediction models and in other settings to improve the accuracy of risk prediction. Considering the early deterioration in the performance of predictive mortality models, especially in terms of calibration, periodic updates are needed. Additionally, further exploration of the clinical validity of the model in the use of funnel plots and EWMA charts is required.

## Supplementary Information

**Additional file 1: Table S1** Coefficients of models 1 and 2. **R code for fitting models 1, 2, and 3. Fig. S1** EWMA charts for the 44 ICUs (recalibrated with the method used in model 3). Sequential admissions are presented on the *x*-axis. The red lines show the exponentially weighted moving average of mortality, with starting points of the average mortality in each ICU during the study period and with a weight of 0.005 on the latest data. The light and dark green lines are the control limits representing two- and three-standard deviations, respectively. EWMA, exponentially weighted moving average; ICU, intensive care unit

**Additional file 2.** Model 3 predicted mortality calculation.

## Data Availability

The authors’ agreement with the JIPAD project does not allow us to publish the data used for this manuscript or to share it with others.

## Abbreviations

AIC: Akaike information criterion
ANZICS: Australian and New Zealand Intensive Care Society
APACHE: Acute Physiology and Chronic Health Evaluation
AUROC: Area under the receiver operating characteristic curve
ICNARC: Intensive Care National Audit & Research Centre
ICU: Intensive care unit
JIPAD: Japanese Intensive Care Patient Database
JROD: Japan Risk of Death
