# Supplementary material for: Development and validation of the predictive risk of death model for adult patients admitted to intensive care units in Japan: an approach to improve the accuracy of healthcare quality measures
Source: J Intensive Care. 2021 Feb 15;9:18. doi: 10.1186/s40560-021-00533-z (PMC7885245; doi:10.1186/s40560-021-00533-z)
Supplement: Supplementary file 1 — Additional file 1: Table S1 Coefficients of models 1 and 2. R code for fitting models 1, 2, and 3. Fig. S1 EWMA charts for the 44 ICUs (recalibrated with the method used in model 3). Sequential admissions are presented on the x-axis. The red lines show the exponentially weighted moving average of mortality, with starting points of the average mortality in each ICU during the study period and with a weight of 0.005 on the latest data. The light and dark green lines are the control limits representing two- and three-standard deviations, respectively. EWMA, exponentially weighted moving average; ICU, intensive care unit [file 40560_2021_533_MOESM1_ESM.docx]

Additional file 1

**Table S1** Coefficients of models 1 and 2

|  | Coefficient | Standard Error | *p* value |
| --- | --- | --- | --- |
| **Model 1** |  |  |  |
| Intercept | −1.33 | 0.025 | < .001 |
|  |  |  |  |
| **Model2** |  |  |  |
| Intercept | −1.32 | 0.026 | < .001 |
| lp | 1.02 | 0.016 | < .001 |

*lp* linear predictor of the APACHE III-j model

**R code for fitting models 1, 2, and 3**

# The dataset is stored as df. “inhospital_death” is a binary variable for in-hospital mortality, where 1 indicates death and 0 indicates alive at hospital discharge.

# “APACHEIIIj_predicted_mortality” is the predicted risk of death calculated by the APACHE III-j model.

# “ICU_ID” is a unique identifier for each participating ICU.

model1 <- glm(inhospital_death ~ offset(log(APACHEIIIj_predicted_mortality/(1 − APACHEIIIj_predicted_mortality))), data = df, family = binomial)

model2 <- glm(inhospital_death ~ log(APACHEIIIj_predicted_mortality/(1 − APACHEIIIj_predicted_mortality)), data = df, family = binomial)

library(mgcv)

model3 <- gam(inhospital_death ~ s(log(APACHEIIIj_predicted_mortality/(1 − APACHEIIIj_predicted_mortality))) + s(ICU_ID, bs="re"), data = df, family = binomial, method = "REML")

**Fig. S1** EWMA charts of the 44 ICUs (recalibrated with the method used in Model 3)


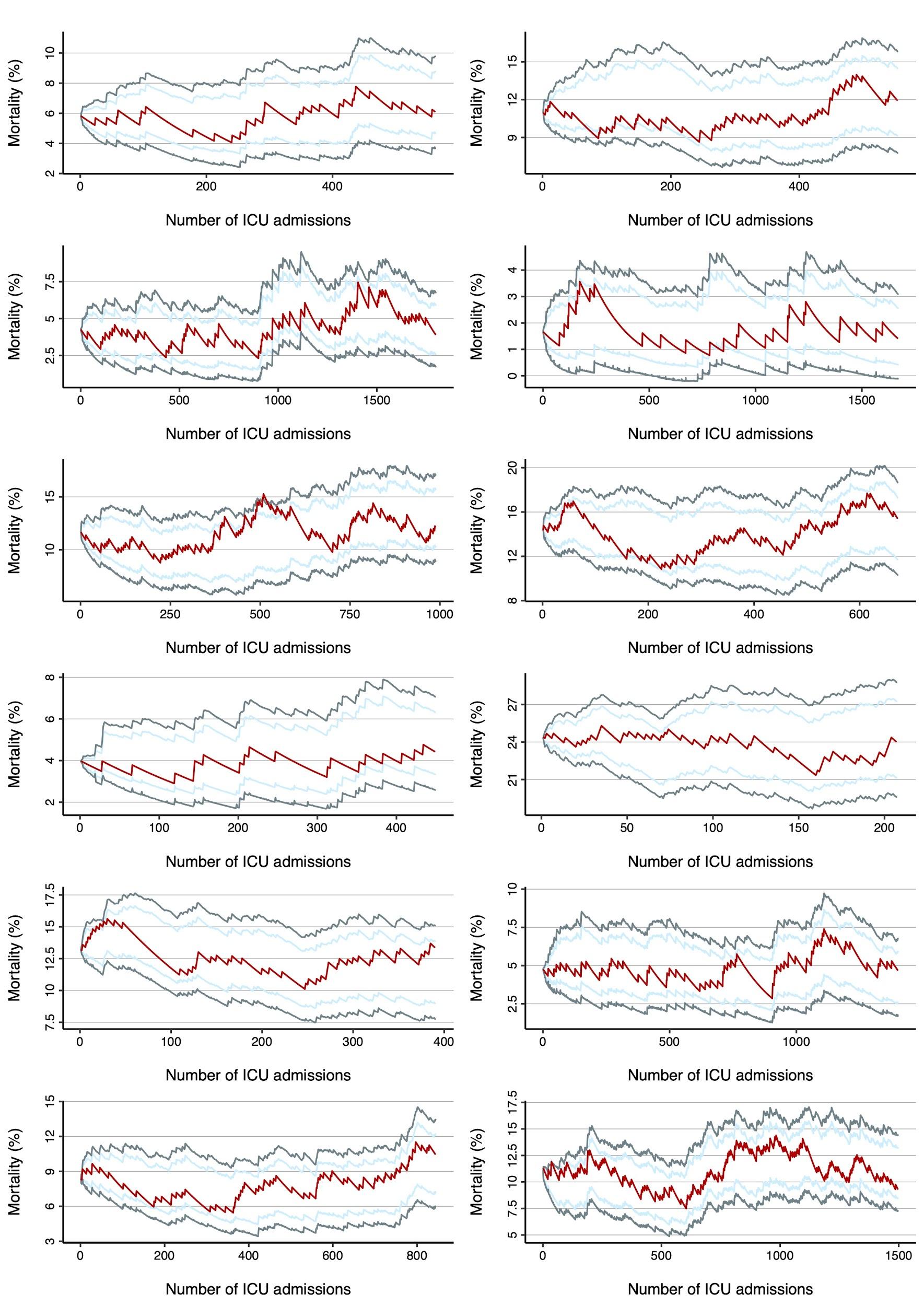

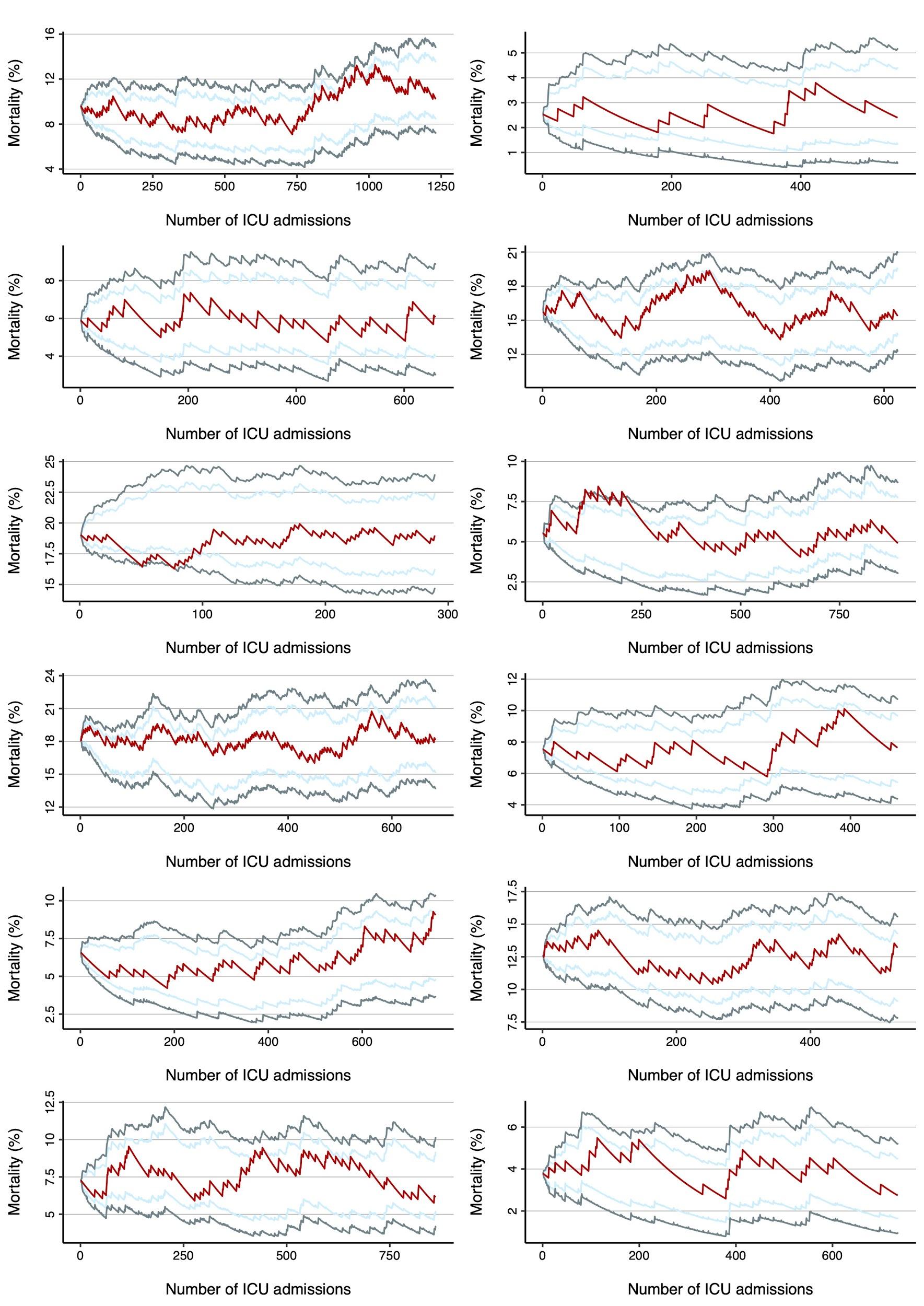

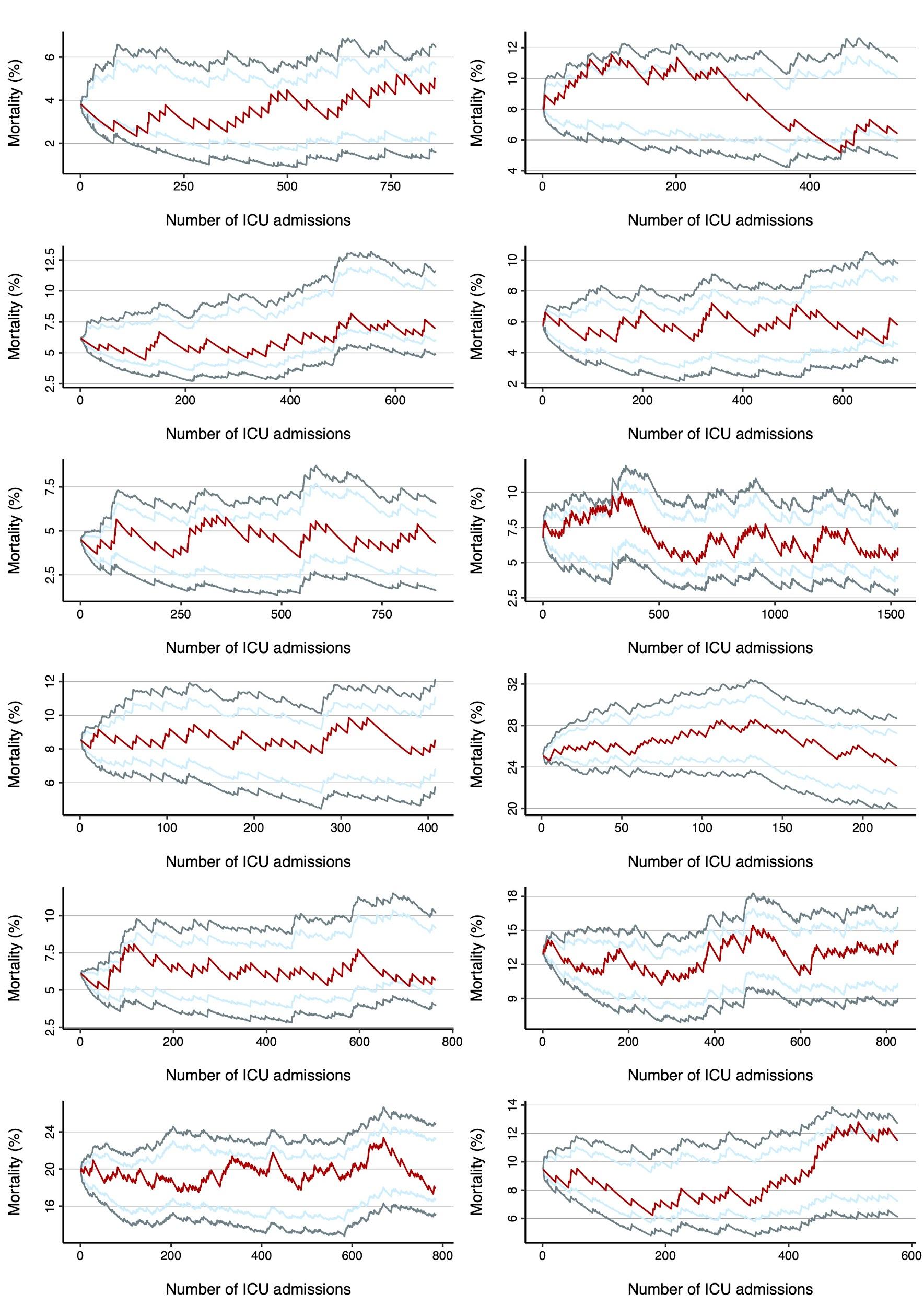


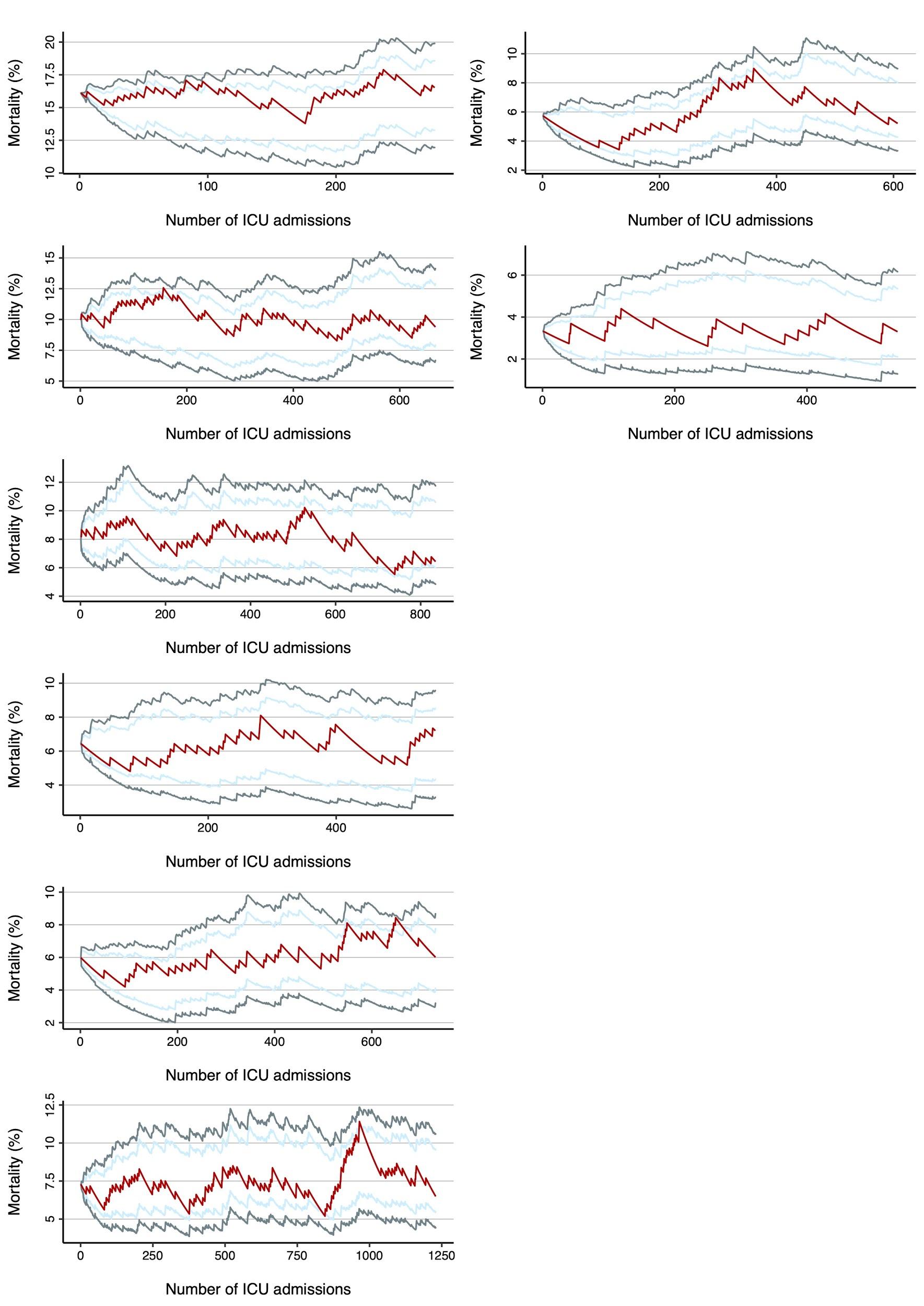


Sequential admissions are presented on the *x*-axis. The red lines show the exponentially weighted moving average of mortality, with starting points of the average mortality in each ICU during the study period and with a weight of 0.005 on the latest data. The light and dark green lines are the control limits representing two- and three-standard deviations, respectively.

EWMA, exponentially weighted moving average; ICU, intensive care unit
